# *Adar* Regulates *Drosophila melanogaster* Spermatogenesis via Modulation of BMP Signaling

**DOI:** 10.3390/ijms25115643

**Published:** 2024-05-22

**Authors:** Qian Zhang, Xinxin Fan, Fang Fu, Yuedan Zhu, Guanzheng Luo, Haiyang Chen

**Affiliations:** 1 MOE Key Laboratory of Gene Function and Regulation, Guangdong Province Key Laboratory of Pharmaceutical Functional Genes, State Key Laboratory of Biocontrol, School of Life Sciences, Sun Yat-sen University, Guangzhou 510275, China; 2 Laboratory of Stem Cell and Aging Research, Frontiers Science Center for Disease-Related Molecular Network, State Key Laboratory of Respiratory Health and Multimorbidity and National Clinical Research Center for Geriatrics, West China Hospital, Sichuan University, Chengdu 610041, China

**Keywords:** *Drosophila*, early spermatogenesis, male infertility, ADAR, BMP signaling

## Abstract

The dynamic process of *Drosophila* spermatogenesis involves asymmetric division, mitosis, and meiosis, which ultimately results in the production of mature spermatozoa. Disorders of spermatogenesis can lead to infertility in males. ADAR (adenosine deaminase acting on RNA) mutations in *Drosophila* cause male infertility, yet the causative factors remain unclear. In this study, immunofluorescence staining was employed to visualize endogenous ADAR proteins and assess protein levels via fluorescence-intensity analysis. In addition, the early differentiation disorders and homeostatic alterations during early spermatogenesis in the testes were examined through quantification of transit-amplifying region length, counting the number of GSCs (germline stem cells), and fertility experiments. Our findings suggest that deletion of ADAR causes testicular tip transit-amplifying cells to accumulate and become infertile in older male *Drosophila*. By overexpressing ADAR in early germline cells, male infertility can be partially rescued. Transcriptome analysis showed that ADAR maintained early spermatogenesis homeostasis through the bone-morphogenetic-protein (BMP) signaling pathway. Taken together, these findings have the potential to help explore the role of ADAR in early spermatogenesis.

## 1. Introduction

Spermatogenesis involves an intricate series of processes within the seminiferous tubules, facilitating the continuous production of male gametes, specifically spermatozoa [1]. In mammals, this dynamic and continuous program spans several months, comprising over 40 distinct stages from spermatogonia to elongated spermatozoa [2]. Direct or indirect factors that disrupt these processes can impair spermatogenesis, ultimately leading to reduced male fertility [3,4,5,6,7,8].

**Figure 1:**
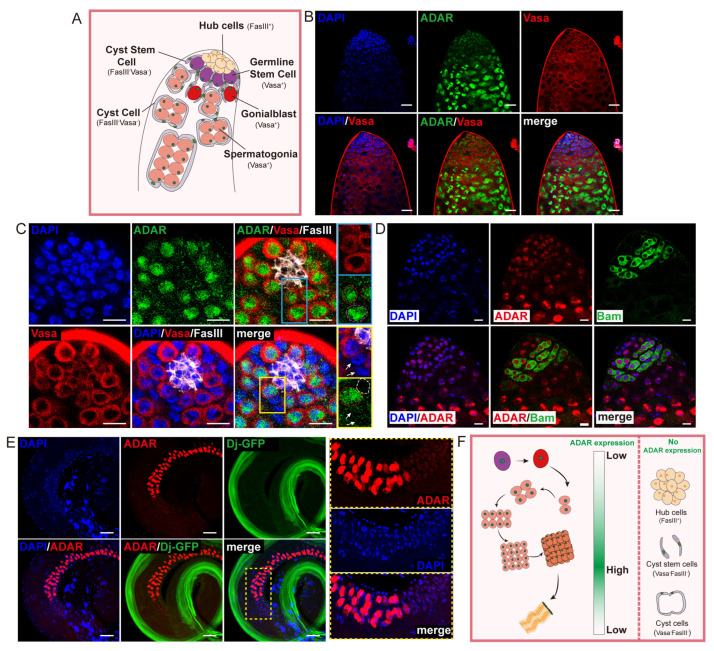
ADAR is highly expressed in *Drosophila* male germline cells. (**A**) Schematic diagram of *Drosophila* testis-tip-cell type. The tip of the testis mainly comprises various cell types, such as hub cells, germline stem cells (GSCs), cyst stem cells (CySCs), gonialblasts (GBs), spermatogonia cells, etc. (**B**) Immunostaining of the apical region of the young testis with ADAR-HA flies. HA (green) indicated ADAR, and Vasa (red) indicated germline cells. DNA was stained with DAPI (in blue). Scale bars represent 25 μm. (**C**) Representative image of the stem-cell niche at the tip of the testis. The blue box area is enlarged on the right, showing a GSC (red dotted line) and a GB (pink dotted line). The yellow box area is enlarged on the right to show a GSC (red dotted line) and a hub cell (white dotted line). The white arrows indicate cyst line cells. FasIII (white) indicated hub cells. HA (green) indicated ADAR, and Vasa (red) indicated germline cells. DNA was stained with DAPI (in blue). Scale bars represent 10 μm. (**D**) Representative image of the testis tip. Bam-GFP (green) indicated spermatogonia. HA (red) indicated ADAR. DNA was stained with DAPI (in blue). Scale bars represent 10 μm. (**E**) Representative image of the young testis. Dj-GFP (green) indicated mature sperm. HA (red) indicated ADAR. The yellow dotted box area is enlarged on the right. DNA was stained with DAPI (in blue). Scale bars represent 50 μm. (**F**) Schematic diagram illustrating that ADAR expression was turned on and off sequentially during spermatogenesis. ADAR was not expressed in hub cells, cyst stem cells, and cyst cells.

The testes of adult *Drosophila* have become an ideal model for studying the mechanisms of spermatogenesis and infertility [9,10,11]. The *Drosophila* testis exhibits a single-ended closed helical tubular configuration characterized by diverse cell types [12]. Spermatogenesis initiates with the mitotic division of germ stem cells located at its tip, followed by sequential processes, including meiosis and sperm morphogenesis. These series of events result in the generation of mature sperm cells, ultimately stored in seminal vesicles (SV) and discharged via the ejaculatory duct (ED) to facilitate mating [12,13]. The maintenance of spermatogenesis involves two distinct populations of stem cells situated at the tip of the testis, germline stem cells (GSCs) and somatic cyst stem cells (CySCs), which are anchored around the hub cells composing the “niche” (Figure 1A) [14,15]. Typically, a pair of CySCs revolves around a GSC. An asymmetric division of a GSC produces two daughter cells, one that remains a self-renewing stemness and the other called a progenitor gonialblast cell (GB). The structure of two somatic cyst cells (Cs) surrounding a GB is called a cyst, followed by four consecutive mitosis and two meiosis to produce 64 spermatid cells, which finally develop into mature sperm cells (Figure S1A). As *Drosophila* ages, the testis undergoes atrophy, accompanied by a reduction in GSC numbers. Despite this decline, the remaining GSCs retain the capacity for self-renewal and differentiation. However, there is a decrease in the number of Bam (bag of marbles)-positive spermatogonia undergoing differentiation [16,17,18,19]. Although the smooth progression of spermatogenesis is essential for male fertility, its mechanism remains unclear.

Adenosine deaminases acting on RNA (ADAR) play a crucial role in regulating cellular homeostasis via post-transcriptional modifications [20,21,22]. Recent investigations unveil novel functions of ADAR1 that are associated with cellular senescence in vitro [20]. In *Drosophila*, only one *Adar* gene is encoded, which provides a favorable opportunity to study more deeply the relationship between *Adar* and aging in vivo. Numerous transcripts modified by ADAR are notably expressed in the central nervous system (CNS). Mutations in *Adar* at synapses manifest as defects in neurotransmission [23,24,25,26] and sleep defects [26], as well as alterations in circadian motor patterns and abnormal male courtship in *Drosophila* [27]. ADAR also governs the differential expression of microRNAs, such as let-7-complex [28], and participates in the behavioral temperature adaptation of *Drosophila* [29]. Loss of editing activity in ADAR leads to innate immune induction and neurodegeneration [30]. ADAR mutations in *Drosophila* have been reported to cause male sterility [25], but it is unclear which stage of spermatogenesis is specifically affected by ADAR and the mechanisms that cause infertility.

Here, we elucidate the significant role of *Adar* in the early stage of spermatogenesis in *Drosophila* males. ADAR exhibits specific expressions within germline cells and increases with aging. Following aging, *Adar* depletion results in the aberrant accumulation of transit-amplifying cells at the testis tip and infertility in *Drosophila*. Transcriptome analysis showed that ADAR functions cell-autonomously to regulate the bone-morphogenetic-protein (BMP) signaling in GSCs. Collectively, these findings unveil a noteworthy contribution of ADAR in early spermatogenesis within the testes.

## 2. Results

### 2.1. ADAR Highly Expressed in Drosophila Male Germline Cells

To determine whether ADAR was necessary for spermatogenesis in male *Drosophila*, we analyzed the expression pattern of the ADAR protein (using the endogenous ADAR-HA *Drosophila* reporter line) in the testes (Figure S1B,C). The immunofluorescence analyses demonstrated the expression pattern of ADAR within the testes of young flies. ADAR was initially observed in the tip region, exhibiting an upregulation in differentiating daughter cells and ultimately becoming inactive in spermatids (Figure S1C). To further observe the specific cell types expressing ADAR in the testis, various cell-specific antibodies were chosen for co-staining with ADAR-HA. ADAR expression was notably confined to the germ cell line expressing the germline-specific marker Vasa (Figure 1A,B). At the apical tip of the testis, ADAR colocalized with GSCs exhibiting high Vasa expression, located adjacent to hub cells that act as a niche. Although no specific marker for CySCs was used, it was determined by antibody-negative expression that ADAR was not expressed in CySCs (Vasa^−^ FasIII^−^) and hub cells (FasIII^+^) (Figure 1A,C). In addition, ADAR was detected in the differentiating GBs that were not directly anchored to the hub and spermatogonia cells expressing the Bam-GFP reporter (Figure 1C,D). Meanwhile, attenuated ADAR expression was observed in early spermatocytes (Figure 1D). Following this stage, ADAR expression sharply increased throughout spermatocyte differentiation (Figure 1B,D). Subsequently, ADAR content exhibited a rapid decline in round spermatids and was completely absent in mature sperm (Dj-GFP^+^) (Figure 1E). These data suggest that ADAR expression was turned on and off sequentially in germline cells during spermatogenesis (Figure 1F).

### 2.2. Adar Loss Suppresses Male-Germline-Cell Differentiation in Drosophila Testis upon Aging

**Figure 2:**
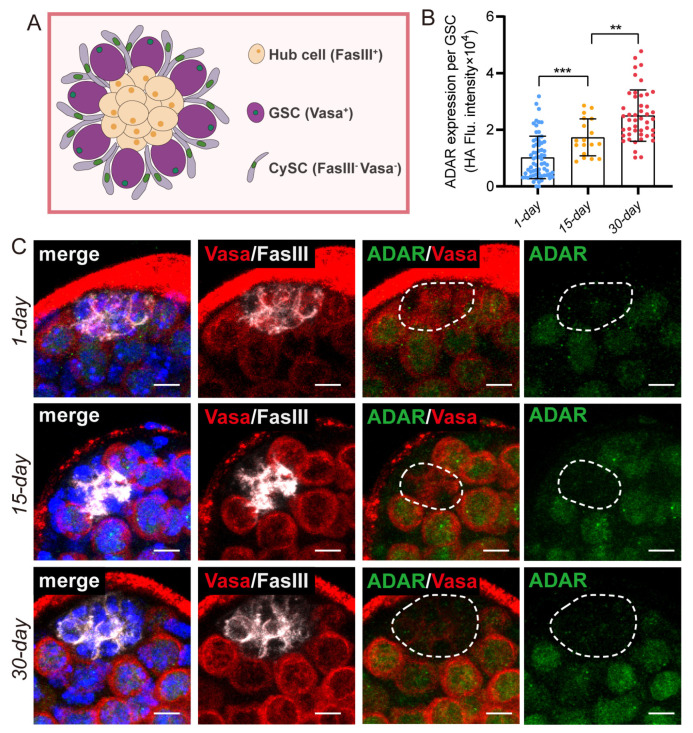
The expression of ADAR increases in GSCs with aging. (**A**) Depiction of the testis tip, illustrating the organization of GSCs and CySCs with the hub cells. (**B**) Quantification of the ADAR protein in Vasa^+^ GSCs of a testis. Each dot corresponds to one GSC. (**C**) Immunofluorescence images of the testis tip of 1-day, 15-day, and 30-day flies. HA (green) indicated ADAR, FasIII (white) indicated hub cells, and Vasa (red) near the hub cell marked GSCs. The white dotted line indicates the testis niche. DAPI stained the nuclei (blue). Scale bars represent 5 μm. ** *p* < 0.01; *** *p* < 0.001.

The *Drosophila* testis becomes thinner with age, and GSCs that distance themselves from the niche exhibit diminished division capability [19]. In subsequent experiments, variations in ADAR protein expression were observed in *Drosophila* GSCs across different ages (1-day, 15-day, and 30-day). We found that the expression of the ADAR protein in the testis’ GSCs gradually increased upon aging (Figure 2A–C). Hence, we hypothesize that ADAR might exert a crucial role in modulating age-related early germ-cell functionality. 

**Figure 3:**
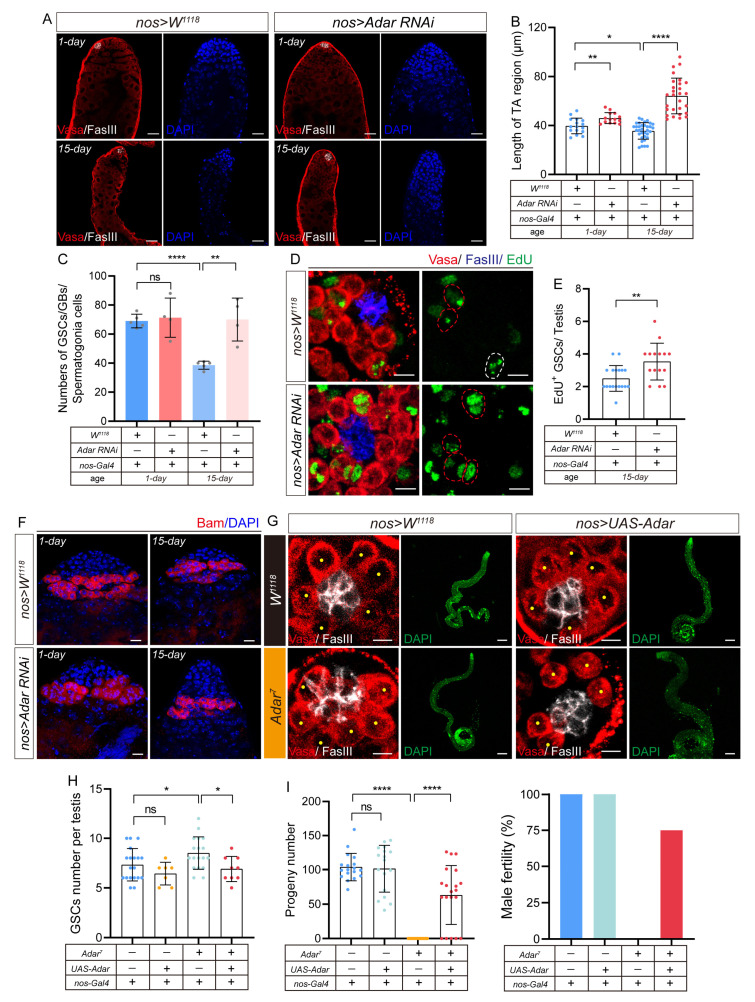
Depletion of ADAR in GSCs leads to the accumulation of transit-amplifying germline cells in the *Drosophila* testis. (**A**) Immunofluorescence images of the testes of flies carrying *nos-Gal4*-driven *W^1118^* (control) and *Adar RNAi*. DAPI-stained bright cells were transit-amplifying regions. DAPI stained the nuclei (blue). FasIII (white) indicated hub cells, and Vasa (red) indicated germline cells. Scale bars represent 25 μm. (**B**) Quantification of the length of TA regions in the testis with indicated genotypes. Flies carrying *nos-Gal4*-driven *W^1118^* were used as controls. Each dot represents a testis. (**C**) Quantification of the number of GSCs, GBs, and spermatogonia in different genotypes of *Drosophila*. (**D**) Representative image of the EdU-labeling experiment in the testis of a 15-day *Drosophila* carrying *nos-Gal4*-driven *W^1118^* (control) and *Adar RNAi*. Cells that stain positive for EdU were in the mitotic S phase. FasIII (white, hub cells) and Vasa (red) near the hub cell marked GSCs. The red dotted line represents the GSCs, and the white dotted line represents non-GSCs. Scale bars represent 5 μm. (**E**) Quantification of the number of EdU-labeled GSCs in the testes of 15-day flies carrying *nos-Gal4*-driven *W^1118^* (control) and *Adar RNAi*. (**F**) Representative image of Bam-HA in the testes of 1-day and 15-day *Drosophila* carrying *nos-Gal4*-driven *W^1118^* (control) and *Adar RNAi*. Bam-HA (red) marked spermatogonia. Scale bars represent 10 μm. (**G**) Immunofluorescence images of a testis tip with Vasa (red) and FasIII (white) staining for *nos-Gal4*-driven *W^1118^* (control), *UAS-Adar*, *Adar^7^*, and *Adar^7^* with *nos-Gal4*-driven *UAS-Adar* in 15-day *Drosophila*. Scale bars represent 5 μm. DAPI (green)-stained-image scale bars represent 100 μm. (**H**) Quantification of the average number of GSCs per testis in different genotypes of flies. (**I**) Mean number of progenies for flies of different genotypes and rate of male fertility. * *p* < 0.05; ** *p* < 0.01; **** *p* < 0.0001; ns, *p* > 0.05.

**Figure 4:**
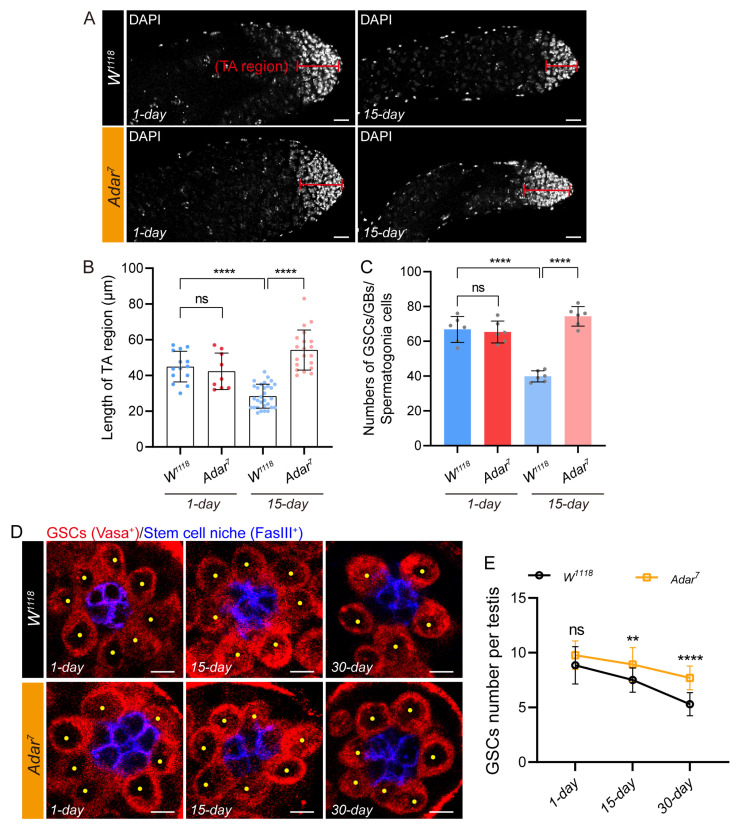
*Adar* loss suppresses male-germline-cell differentiation in the *Drosophila* testis upon aging. (**A**) Immunofluorescence images of the testes of flies with and without *Adar*. DAPI-stained bright cells were transit-amplifying (TA) regions. DAPI stained the nuclei (white). Scale bars represent 20 μm. (**B**) Quantification of the length of TA regions in the testis of *W^1118^* (wild-type) and *Adar* mutant flies at different ages. (**C**) Quantification of the number of GSCs, GBs, and spermatogonia in different genotypes of *Drosophila*. (**D**) Immunofluorescence images of different-age testis tips of *W^1118^* and *Adar* mutant flies. FasIII (blue) indicated hub cells, and Vasa (red) near the hub cell marked GSCs. A yellow dot indicates a GSC. DAPI stained the nuclei (blue). Scale bars represent 5 μm. (**E**) Average number of GSCs per testis. ** *p* < 0.01; **** *p* < 0.0001; ns, *p* > 0.05.

To validate the above conjecture, we constructed an *Adar* mutant *Drosophila* strain. Consistent with the previous report [25], we observed that the *Adar^7^* mutant exhibited conditional lethality. Despite the ability of *Adar^7^* male mutants to develop into morphologically normal adults akin to *wild-type* males, their number was notably reduced, owing to recessive behavioral defects. Moreover, due to male sterility in *Adar^7^* mutants (Figure 3I), we were unable to obtain homozygous *Adar^7^* mutant females. We first examined the squashed testis sample, and the morphology of the testis of the *Adar* mutant was not much different from the control under phase-contrast microscopy (Figure S1D,E). Then, we compared the TA (transit-amplifying) division region length of young and aged *Adar* mutant males to age-matched controls (Figure 4A). In contrast to the *W^1118^*, aging *Adar* mutant testes exhibited an abnormal accumulation presence of early germ cells prominently stained with DNA dyes, while no distinction was observed when younger (Figure 4A,B). The decline in the quantity of GSCs and Bam-positive spermatogonia during aging ultimately leads to a shortening of the TA region of the senescent testes [16,31]. During aging, the total number of GSCs and spermatogonia in *Adar* mutants was significantly higher than that of wild-type *Drosophila* (Figure 4C). We hypothesize that the accumulation of the older testis TA cells caused by *Adar* mutations was attributable to the preservation of GSC stemness. Consistent with previous studies [16], the number of GSCs that were physically attached to the hub in the *W^1118^* testis exhibited a decline with advancing age (Figure 4D,E). In newborn one-day-old male flies, an average of 8.8 GSCs per testis was observed. By 15 days, this number decreased to 7.5 GSCs per testis. In 30-day flies, there was a notable reduction, with an average of 5.2 GSCs per testis. Interestingly, the number of GSCs in *Adar* mutants surpassed that of *W^1118^* flies starting from day 15 and continued to be consistently higher until day 30 (Figure 4D,E). At 15 days of age, *Adar* mutants exhibited an average of 8.9 GSCs per testis. By the 30th day, the average number of GSCs in the testis of *Adar* mutant *Drosophila* was 7.7 (Figure 4D,E). Consequently, the *Adar* mutant may induce stemness maintenance of GSCs and early germ-cell accumulation by preventing the differentiation of testicular germ cells in aging *Drosophila*.

### 2.3. Depletion of ADAR in GSCs Leads to the Accumulation of Transit-Amplifying Germline Cells in Drosophila Testis

**Figure 5:**
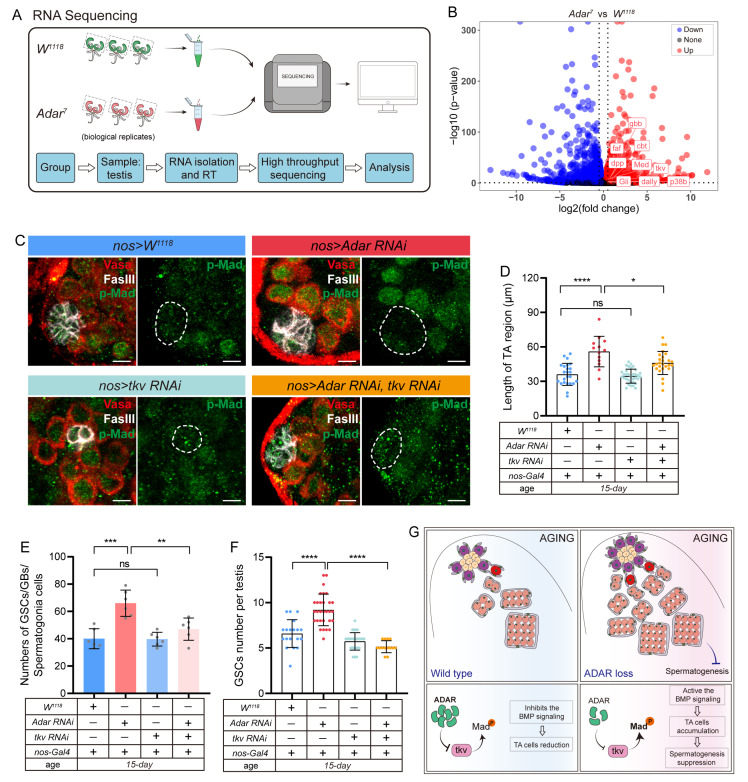
*Adar* regulates male-germline-stem- and progenitor-cell differentiation through modulation of BMP signaling. (**A**) Schematic diagram of the RNA-seq sample preparation and process. (**B**) Volcano plots of differentially expressed genes in a pair-wise comparison of *W^1118^* (control) and *Adar^7^*. (**C**) Immunofluorescence images of the testis tips of flies carrying *nos-Gal4*-driven *W^1118^* (control), *Adar RNAi*, *tkv RNAi*, and *Adar RNAi* with *tkv RNAi* in 15 days. Scale bars represent 10 μm. P-Mad (green), FasIII (white, hub cells), and Vasa (red) near the hub cell marked GSCs. The white dotted line indicates the testis niche. (**D**) Quantification of the length of TA regions in the testis of different genotype flies in 15 days. (**E**) Quantification of the number of GSCs, GBs, and spermatogonia in different genotypes of *Drosophila*. (**F**) Quantification of the average number of GSCs per testis in different-genotype flies. (**G**) Schematic diagram of ADAR modulation of the BMP signaling pathway in the testis of aging *Drosophila*. * *p* < 0.05; ** *p* < 0.01; *** *p* < 0.001; **** *p* < 0.0001; ns, *p* > 0.05.

To determine whether the abnormal accumulation of TA cells in *Adar* mutants was due to the lack of *Adar* in GSCs, we performed *nanos-Gal4* (*nos-Gal4*) drivers exhibiting specific activity in GSCs to induce *Adar* depletion. We found that depletion of *Adar* specifically in the GSCs (by *nos-Gal4*) but not CySCs (by *C587-Gal4*) caused amplification of the TA region on both day 1 and day 15, which confirmed the previous hypothesis (Figure 3A–C and Figure S2A,D–F). Unexpectedly, the testes that lost *Adar* remained small (Figure 3A), and fertility declined (Figure S2B,C), corresponding to age-matched controls. Moreover, our analysis indicated that in comparison to controls, aged males with ADAR depletion maintained a higher number of GSCs within their niche (Figure 5F). While the removal of ADAR increased the GSC count within the aging testis, it remains uncertain whether these GSCs can engage in normal self-renewal processes. To investigate this, we conducted immunofluorescent staining of the testis using EdU (5-ethynyl-2′-deoxyuridine) as a marker to label GSCs undergoing mitosis. EdU was a base analog that could be added as a substrate to DNA being synthesized to identify cells in the S phase [32]. The average number of EdU-labeled GSCs in 15-day-old control flies was 2.64, while the average number of EdU-labeled GSCs in 15-day-old flies from *nos-Gal4*-driven *Adar RNAi* was 3.27 (Figure 3D,E). These experiments demonstrated that GSCs lacking ADAR retained the capacity for normal mitotic activity, exhibiting a significantly higher number of GSCs undergoing mitosis compared to aging wild-type *Drosophila*. Then, we noted an increase in the distance of the Bam expression region from the tip upon ADAR loss in the aging testis (Figure 3F). In conclusion, these results suggest that ADAR depletion in GSCs leads to the abnormal accumulation of TA cells upon aging.

Similarly, we observed that the maintenance of the *Adar* mutant GSCs did not lead to an improvement in the testis aging morphology at 15 days (Figure 3G,H). Analogous to the age-matched control, the testes of the aging *Adar* mutant remained small and thin (Figure 3G). Overexpression of *Adar* cDNA in *Adar* mutants reinstated the GSC count to a level comparable to that of control males (Figure 3G,H) and partially rescued the sterile phenotype of the mutant flies (Figure 3I). Consequently, it demonstrated that early germ cells that lose *Adar* may accumulate at the TA stage due to differentiation disorders, rendering these cells incapable of sustaining spermatogenesis in aging males.

### 2.4. Adar Regulates Male-Germline-Stem- and Progenitor-Cell Differentiation through Modulation of BMP Signaling

To identify the mechanism with which ADAR regulates the germline-stem- and progenitor-cell differentiation during testis aging, RNA sequencing (RNA-seq) analyses were performed on dissected testes from the *W^1118^* (control) and *Adar^7^* flies (Figure 5A and Figure S3A,B) (results listed in Table S1). The volcano plot shows the increased expression of genes related to the BMP signaling pathway, such as *dpp*, *gbb*, *Med*, etc., (Figure 5B). The initiation of the BMP signaling pathway is imperative for suppressing GSC premature differentiation and preserving its stem-cell attributes [33,34]. Depletion of the BMP-signaling-pathway constituents in GSCs results in aberrant pathway activity, premature differentiation, and a notable reduction in the GSC population [14,35]. To confirm the results of the RNA-seq analyses, real-time quantitative PCR (RT-qPCR) analyses were performed using indicated genotypes of testicular tissue. The results of RT-qPCR analyses of the selected genes showed similar expression patterns to those of RNA-seq analysis during aging (Figure S3C).

Hence, we investigated whether GSCs lacking *Adar* could be sustained through BMP signaling, assessing the phosphorylation status of the transcription factor Mad (Mothers against Dpp), a hallmark of pathway activation [36]. Compared to the control testes, heightened p-Mad was observed in GSCs surrounding the hub cells following *Adar* knockdown driven by *nos-Gal4* upon aging (Figure 5C). Furthermore, we found that attenuation of BMP activity in GSCs by *nos-Gal4*-driven *thickveins* (*tkv*) *RNAi* significantly rescued the nuclear accumulation of p-Mad in testes carrying *nos-Gal4*-driven *Adar RNAi* (Figure 5C). Simultaneously, we noted that the inhibition of the BMP signaling pathway markedly corrected the accumulation of the TA region and reduced the GSC count during testicular aging induced by *Adar* knockdown (Figure 5D–F). Consequently, ADAR governs germ-cell-lineage maintenance and development by modulating BMP signaling (Figure 5G).

## 3. Discussion

Recent investigations employing diverse biological models have established a close correlation between germline cell behavior and the progression of aging [37,38,39,40]. Nevertheless, the precise mechanisms governing the function of germline cells remain incompletely elucidated. Evidence exists linking *Drosophila* ADAR to male infertility [25] and neurodegenerative disorders [24]. This study presents a significant revelation regarding the contribution of the ADAR protein to the development of the male reproductive lineage in *Drosophila*. Our findings underscore the essential role of ADAR in the initial phases of spermatogenesis. We noted an elevation in ADAR expression in aging testes, and the manipulation of ADAR expression reduced through genetic interventions during the aging process resulted in germ-cell-differentiation disorders, elevated numbers of GSCs, and accumulation of TA cells, ultimately leading to male infertility.

This study advances our understanding of the function of the RNA-editing enzyme ADAR in *Drosophila* testicular GSCs. Our findings indicate that ADAR expression is at the tip of the testis, further confirming its specific expression in germline cells, including GSCs. Notably, ADAR mutations lead to sterility in male *Drosophila.* Early germ-cell accumulation in the testes impairs spermatogenesis in aged flies. Overexpression of full-length ADAR in GSCs exhibits the partial rescue of the sterility of *Adar* mutants, suggesting the involvement of ADAR in GSC functionality.

Aging disrupts tissue homeostasis, which necessitates the support of tissue-specific stem cells [41,42,43]. *Drosophila* constitutes an exemplary model for investigating age-associated alterations in stem-cell functionality. Both cellular autonomy and extrinsic stimulation play pivotal roles in influencing stem-cell activity. Two stem-cell lineages, GSCs and CySCs, are present at the apical tip of the testis in *Drosophila* [44,45]. Our investigation reveals spatiotemporal-specific differential expression of ADAR in GSCs. ADAR was specifically expressed in GSCs and increases with aging. The absence of ADAR in age-related GSCs was associated with a substantial increase in the number of testicular GSCs and an accumulation of the TA region. We posit that the decrease in ADAR expression signifies a shift in GSC functionality, thereby contributing to the accumulation of GSCs over time, and that the increased GSCs retain the capacity for mitotic activity. All these findings distinctly revealed that ADAR functions cell-autonomously in GSCs to maintain early spermatogenesis. Previous reports indicated that an age-related elevation in miR-9a levels may impede stem-cell accumulation at the expense of spermatogenesis [19]. It was reasonable to speculate that ADAR deletion impairs spermatogenesis in aged *Drosophila* by hindering differentiation, leading to early germ cell accumulation. The association between ADAR and germ-lineage-cell differentiation will be a focal point of our forthcoming research. ADAR exhibits additional functionalities independent of RNA editing [30]. Reports suggest that ADAR is implicated in the regulation of cell senescence in vitro, independent of its RNA-editing function [20]. Consequently, whether the regulation of germline cells by ADAR is contingent on its RNA editing ability will be a central focus of our continued exploration.

The BMP signaling pathway plays a crucial role in germ stem cells maintaining and renewing [36,46,47]. Mad represents a pivotal downstream target within the BMP pathway, wherein pathway activation induces Mad phosphorylation (p-Mad) and accumulation within the cell nucleus [48,49]. In this study, we investigated the interplay between ADAR and BMP signaling during TA division in *Drosophila* testicular spermatogonia. Transcriptome analysis unveiled significant activation of the BMP signaling pathway in the ADAR-depletion-aging testis, and the expression of p-Mad was increased in the IF assay. Subsequent experiments showed that inhibiting the BMP signaling pathway in senescent GSCs could counteract the abnormal accumulation of TA cells caused by ADAR deletion. This highlights the potential of ADAR as a target for exploring the mechanisms of early spermatogenesis.

In summary, we found an elevation in ADAR expression with aging in the *Drosophila* testis. Specific knockdown of *Adar* in aging germline cells demonstrated abnormal accumulation of TA cells was achieved by upregulating the BMP signaling pathway. Simultaneously, the overexpression of ADAR was observed to rescue the sterile phenotype in older *Adar* mutant flies partially. These findings underscore the pivotal role of ADAR in early spermatogenesis within the testes of *Drosophila* (Figure 5G). We investigated the function of ADAR in maintaining germ-cell differentiation and germ-stem-progenitor-cell division, elucidating the correlation between ADAR and BMP signaling in the *Drosophila* testis. Our findings robustly indicate that ADAR controls normal germline-cell differentiation through germline BMP signaling. This research contributes to the broadening of our comprehension regarding early spermatogenesis and the homeostasis of the germline.

## 4. Materials and Methods

### 4.1. Drosophila Stocks

This study was approved by the Experimental Animal Ethics Committee of Sichuan University of West China Hospital (Approval No. 20231225007). Flies were housed on freshly prepared standard cornmeal agar medium. Generally, flies were housed in incubators with normal light and dark cycles at 25 °C. The flies were anesthetized with carbon dioxide and then dissected in PBS. Only the testes of adult male flies were used for experiments. Wild-type flies were *W^1118^*. Additional strains used were *nanos-Gal4* (Bloomington *Drosophila* Stock Center (BDSC) 4937); *C587-Gal4* (from Xin Chen); *tub-Gal4/TM3.sb* (from Allan Spradling); *Adar RNAi* (TsingHua Fly Center, THU2946); *tkv RNAi* (Vienna *Drosophila* Resource Center (VDRC) 3059); *Bam-GFP* and *Bam-HA* (from Xin Chen); and *Dj-GFP* (BDSC 5417).

### 4.2. Generation of Knock-In, Knock-Out, and Transgenic Fly Lines

Knock-in experiments were performed using our in-house manufactured *Adar-HA* reporter line. The final vector was validated by sequencing and injected by Fungene Biotechnology (Nanjing, China). The methodology was previously reported [50]. The sgRNA was designed using an online software application (http://targetfinder.flycrispr.neuro.brown.edu/, accessed on 9 October 2023) as follows:

Target1-*Adar*-sgRNA: 

Forward: 5′-CCGAACTCGTCTTGTTCAAT-3′

Reverse: 5′-ATTGAACAAGACGAGTTCGG-3′

Target2-*Adar*-sgRNA: 

Forward: 5′-TAGTGCTGAGTGCAGTCATT-3′

Reverse: 5′-AATGACTGCACTCAGCACTA-3′.

The transgenic line that we made in-house was *UAS-Adar-HA*. The methodology was previously reported [50]. The *Adar* cDNA primers were as follows:

*Adar* L: 5′-ATGACACTCTGCCGATACA-3′

*Adar* R: 5′-TTCGGCAAGACCGAACT-3′.

The knock-out line that we made in-house was *Adar^7^*, and the final vectors were verified by sequencing. The methodology was previously reported [50]. The sgRNAs were again designed using an online software application (http://targetfinder.flycrispr.neuro.brown.edu/, accessed on 11 March 2023):

Target 1-*Adar*-sgRNA:

5′-ATGTCTTAGCTCATTCAGCA-3′

Target 2-*Adar*-sgRNA:

5′-GTGAATAGAGGTGCGTGTAC-3′.

### 4.3. Immunofluorescence

The testes of adult *Drosophila* were dissected in ice-cold phosphate-buffered saline (PBS); placed in 200 μL of 4% paraformaldehyde (PFA) fixation solution for 0.5 h at room temperature; washed twice with PBST (1% Triton X-100), followed by 5% BSA (1% PBST configuration); and blocked for 1 h at room temperature. The tissues were incubated overnight at 4 °C with primary antibodies dissolved in 1% PBST. After that, they were washed 3 times with 1% PBST for 5 min each before incubating with secondary antibodies and DAPI for 2 h. Following the incubation with secondary antibodies, the testes were washed 3 times with 1% PBST for 5 min each. The primary antibodies for immunofluorescence were as follows: One was rabbit anti-HA (1:1000, Cell Signaling Technology, Danvers, MA, USA, Cat#3724). Mouse anti-FasIII (1:100) and rat anti-Vasa (1:10) were obtained from the Developmental Studies Hybridoma Bank (Iowa City, IA, USA). The others were rabbit anti-Smad3 (phospho S423 + S425) (1:250, abcam, Cambridge, UK, ab52903) and chicken anti-GFP (1:2000, abcam, Cambridge, UK, ab13970). Secondary antibodies were obtained from Invitrogen (Carlsbad, CA, USA), in which the dilution concentration was 1:2000. The image was taken with the Leica TCS-SP8 instrument (Wetzlar, Germany).

### 4.4. Fluorescence-Intensity Statistics

Statistical calculations of the fluorescence intensity of cells of interest were performed on immunofluorescence imaging results obtained by confocal microscopy using the ImageJ software (https://imagej.nih.gov/ij/, accessed on 27 November 2023), as described in a previous report [50].

### 4.5. EdU Labeling Experiments

Adult *Drosophila* testes were dissected and placed in a six-well plate with 200 µL of 4 °C PBS. In brief, 2× of EdU working solution (Beyotime, Shanghai, China, C0081S) was prepared and added to the plate at a final concentration of 10 μM (1×). The incubation occurred for 2 h at room temperature (EdU labeling), followed by fixation with 4% PFA for 15 min and three washes with PBS containing 3% BSA for 5 min each. Next, the testes were incubated with 0.3% PBST for 10 min, followed by another wash with PBS containing 3% BSA for 5 min. The click-reaction solution (Beyotime, Shanghai, China, C0081S) was then added and incubated for 30 min at room temperature in the dark. The testes were then washed 3 times with PBS containing 3% BSA for 5 min each. Subsequently, the incubation of primary and secondary antibodies was performed by standard immunofluorescence procedures.

### 4.6. Fertility Assays

To assess the fertility of male *Drosophila*, a single adult male was selected to mate with three virgin females of the wild type in individual vials containing the standardized medium for *Drosophila*. Unless otherwise stated, the parental flies were transferred to new fresh vials after 9 days of mating at 25 °C. The offspring generation was determined by counting the offspring in both the initial vial and the first subsequent transfer vial.

### 4.7. RNA-Seq

Dissected *Drosophila* testicular tissue was frozen with liquid nitrogen, and subsequently, the entire sequencing was performed by Berry Genomics (Beijing, China). The sequencing platform was based on Novaseq 6000 (Illumina, San Diego, CA, USA), with 150 bp paired-end runs resulting in over 20 million reads per sample. Initial quality control processing was applied to raw RNA-seq data, including filtering out low-quality read and trimming adapter sequences using Trimmomatic (v 0.39). Trimmed RNA-seq reads were aligned to the *Drosophila* reference sequence Ensembl Build BDGP6 (https://ftp.ensembl.org/pub/current_gtf/drosophila_melanogaster/Drosophila_melanogaster.BDGP6.46.112.gtf.gz, accessed on 20 November 2023) using STAR (v2.7.8). FeatureCounts (v2.0.6) was used to calculate the raw gene count matrix for each sample. Differential-expression genes were determined using the R package DESeq2 (v 1.38.3) with default parameters. Genes were considered differentially expressed if the adjusted p-value after Benjamini correction was <0.05 (DESeq2’s default parameters). These data are available in the SRA under the BioProject ID PRJNA1050604.

### 4.8. RNA Extraction and RT-qPCR

Testicular tissue from approximately 30 flies was dissected and placed into an RNA-easy Isolation Reagent (Vazyme, Nanjing, China, R701), followed by RNA extraction according to the kit’s protocol. cDNA was synthesized using the ExonScript RT SuperMix with dsDNase (Exongen, Chengdu, China) and then diluted 5-fold with water for RT-qPCR (real-time quantitative PCR). RT-qPCR was executed on a CFX96TM Real-time PCR System (BIO-RAD, Hercules, CA, USA) employing SYBR qPCR Master Mix (Vazyme, Nanjing, China, Q711) for each sample. Expression values were computed using the 2^−ΔΔCT^ method, and the relative expression was normalized to *Rp49*. The expression in the control sample was further normalized to 1. Primer sequences used for RT-qPCR were as follows: *Rp49* forward primer, 5′-ATCGGTTACGGATCGAACAAGC-3′, and reverse primer, 5′-GTAAACGCGGTTCTGCATGAGC-3′; *dpp* forward primer, 5′-GCCAACACAGTGCGAAGTT-3′, and reverse primer, 5′-ACCACCTGTTGACTGAGTGC-3′; *gbb* forward primer, 5′-CTGGATCATCGCACCAGAGG-3′ and reverse primer, 5′-GTCTGGACGATCGCATGGTT-3′.

### 4.9. Statistics

Statistical analysis was conducted utilizing GraphPad Prism 8.0 (https://www.graphpad.com/, accessed on 9 January 2024). Unless specified otherwise, data were expressed as standard deviation (SD) ± mean and were obtained from a minimum of three independent biological replicates. Statistical significance was assessed using the two-tailed Student’s *t*-test, unless otherwise specified. Significance was denoted in the text or legend, where *p* < 0.05 was deemed statistically significant. See the legend for additional information.

## Data Availability

The obtained RNA-Seq is available in the NCBI Sequence Read Archive under the BioProject ID PRJNA1050604.

## Supplementary Materials

The following supporting information can be downloaded at https://www.mdpi.com/article/10.3390/ijms25115643/s1.

## References

[1] Neto F.T., Bach P.V., Najari B.B., Li P.S., Goldstein M. (2016). Spermatogenesis in humans and its affecting factors. Semin. Cell Dev. Biol..

[2] Bettegowda A., Wilkinson M.F. (2010). Transcription and post-transcriptional regulation of spermatogenesis. Philos. Trans. R. Soc. Lond. B Biol. Sci..

[3] Dura M., Teissandier A., Armand M., Barau J., Lapoujade C., Fouchet P., Bonneville L., Schulz M., Weber M., Baudrin L.G. (2022). DNMT3A-dependent DNA methylation is required for spermatogonial stem cells to commit to spermatogenesis. Nat. Genet..

[4] Huang Q., Liu Y., Zhang S., Yap Y.T., Li W., Zhang D., Gardner A., Zhang L., Song S., Hess R.A. (2021). Autophagy core protein ATG5 is required for elongating spermatid development, sperm individualization and normal fertility in male mice. Autophagy.

[5] Lord T., Nixon B. (2020). Metabolic Changes Accompanying Spermatogonial Stem Cell Differentiation. Dev. Cell.

[6] Pereira C.D., Serrano J.B., Martins F., da Cruz E.S.O.A.B., Rebelo S. (2019). Nuclear envelope dynamics during mammalian spermatogenesis: New insights on male fertility. Biol. Rev. Camb. Philos. Soc..

[7] Yin H., Kang Z., Zhang Y., Gong Y., Liu M., Xue Y., He W., Wang Y., Zhang S., Xu Q. (2021). HDAC3 controls male fertility through enzyme-independent transcriptional regulation at the meiotic exit of spermatogenesis. Nucleic Acids Res..

[8] Eisenberg M.L., Esteves S.C., Lamb D.J., Hotaling J.M., Giwercman A., Hwang K., Cheng Y.S. (2023). Male infertility. Nat. Rev. Dis. Primers.

[9] Sênos Demarco R., Jones D.L. (2020). EGFR signaling promotes basal autophagy for lipid homeostasis and somatic stem cell maintenance in the Drosophila testis. Autophagy.

[10] Witt E., Benjamin S., Svetec N., Zhao L. (2019). Testis single-cell RNA-seq reveals the dynamics of de novo gene transcription and germline mutational bias in Drosophila. eLife.

[11] Gubala A.M., Schmitz J.F., Kearns M.J., Vinh T.T., Bornberg-Bauer E., Wolfner M.F., Findlay G.D. (2017). The Goddard and Saturn Genes Are Essential for Drosophila Male Fertility and May Have Arisen De Novo. Mol. Biol. Evol..

[12] Zhang Z., Pan C., Zhao Y. (2013). Hedgehog in the Drosophila testis niche: What does it do there?. Protein Cell.

[13] White-Cooper H., Bausek N. (2010). Evolution and spermatogenesis. Philos. Trans. R. Soc. Lond. B Biol. Sci..

[14] Leatherman J.L., Dinardo S. (2010). Germline self-renewal requires cyst stem cells and stat regulates niche adhesion in Drosophila testes. Nat. Cell Biol..

[15] Lehmann R. (2012). Germline stem cells: Origin and destiny. Cell Stem Cell.

[16] Boyle M., Wong C., Rocha M., Jones D.L. (2007). Decline in self-renewal factors contributes to aging of the stem cell niche in the Drosophila testis. Cell Stem Cell.

[17] Herrera S.C., de la Maza D.S., Grmai L., Margolis S., Plessel R., Burel M., O’Connor M., Amoyel M., Bach E.A. (2021). Proliferative stem cells maintain quiescence of their niche by secreting the Activin inhibitor Follistatin. Dev. Cell.

[18] Toledano H., D’Alterio C., Czech B., Levine E., Jones D.L. (2012). The let-7-Imp axis regulates ageing of the Drosophila testis stem-cell niche. Nature.

[19] Epstein Y., Perry N., Volin M., Zohar-Fux M., Braun R., Porat-Kuperstein L., Toledano H. (2017). miR-9a modulates maintenance and ageing of Drosophila germline stem cells by limiting N-cadherin expression. Nat. Commun..

[20] Hao X., Shiromoto Y., Sakurai M., Towers M., Zhang Q., Wu S., Havas A., Wang L., Berger S., Adams P.D. (2022). ADAR1 downregulation by autophagy drives senescence independently of RNA editing by enhancing p16(INK4a) levels. Nat. Cell Biol..

[21] Herzner A.M., Khan Z., Van Nostrand E.L., Chan S., Cuellar T., Chen R., Pechuan-Jorge X., Komuves L., Solon M., Modrusan Z. (2021). ADAR and hnRNPC deficiency synergize in activating endogenous dsRNA-induced type I IFN responses. J. Exp. Med..

[22] Stellos K., Gatsiou A., Stamatelopoulos K., Matic L.P., John D., Lunella F.F., Jaé N., Rossbach O., Amrhein C., Sigala F. (2016). Adenosine-to-inosine RNA editing controls cathepsin S expression in atherosclerosis by enabling HuR-mediated post-transcriptional regulation. Nat. Med..

[23] Maldonado C., Alicea D., Gonzalez M., Bykhovskaia M., Marie B. (2013). Adar is essential for optimal presynaptic function. Mol. Cell Neurosci..

[24] Khan A., Paro S., McGurk L., Sambrani N., Hogg M.C., Brindle J., Pennetta G., Keegan L.P., O’Connell M.A. (2020). Membrane and synaptic defects leading to neurodegeneration in Adar mutant Drosophila are rescued by increased autophagy. BMC Biol..

[25] Palladino M.J., Keegan L.P., O’Connell M.A., Reenan R.A. (2000). A-to-I pre-mRNA editing in Drosophila is primarily involved in adult nervous system function and integrity. Cell.

[26] Robinson J.E., Paluch J., Dickman D.K., Joiner W.J. (2016). ADAR-mediated RNA editing suppresses sleep by acting as a brake on glutamatergic synaptic plasticity. Nat. Commun..

[27] Jepson J.E.C., Savva Y.A., Yokose C., Sugden A.U., Sahin A., Reenan R.A. (2011). Engineered alterations in RNA editing modulate complex behavior in Drosophila: Regulatory diversity of adenosine deaminase acting on RNA (ADAR) targets. J. Biol. Chem..

[28] Chawla G., Sokol N.S. (2014). ADAR mediates differential expression of polycistronic microRNAs. Nucleic Acids Res..

[29] Buchumenski I., Bartok O., Ashwal-Fluss R., Pandey V., Porath H.T., Levanon E.Y., Kadener S. (2017). Dynamic hyper-editing underlies temperature adaptation in Drosophila. PLoS Genet..

[30] Deng P., Khan A., Jacobson D., Sambrani N., McGurk L., Li X., Jayasree A., Hejatko J., Shohat-Ophir G., O’Connell M.A. (2020). Adar RNA editing-dependent and -independent effects are required for brain and innate immune functions in Drosophila. Nat. Commun..

[31] Leatherman J.L., Dinardo S. (2008). Zfh-1 controls somatic stem cell self-renewal in the Drosophila testis and nonautonomously influences germline stem cell self-renewal. Cell Stem Cell.

[32] Liu F., Zhang X., Peng Y., Zhang L., Yu Y., Hua P., Zhu P., Yan X., Li Y., Zhang L. (2021). miR-24 controls the regenerative competence of hair follicle progenitors by targeting Plk3. Cell Rep..

[33] Kawase E., Wong M.D., Ding B.C., Xie T. (2004). Gbb/Bmp signaling is essential for maintaining germline stem cells and for repressing bam transcription in the Drosophila testis. Development.

[34] Schulz C., Kiger A.A., Tazuke S.I., Yamashita Y.M., Pantalena-Filho L.C., Jones D.L., Wood C.G., Fuller M.T. (2004). A misexpression screen reveals effects of bag-of-marbles and TGF beta class signaling on the Drosophila male germ-line stem cell lineage. Genetics.

[35] Inaba M., Buszczak M., Yamashita Y.M. (2015). Nanotubes mediate niche-stem-cell signalling in the Drosophila testis. Nature.

[36] Zheng Q., Wang Y., Vargas E., DiNardo S. (2011). magu is required for germline stem cell self-renewal through BMP signaling in the Drosophila testis. Dev. Biol..

[37] Alfano M., Tascini A.S., Pederzoli F., Locatelli I., Nebuloni M., Giannese F., Garcia-Manteiga J.M., Tonon G., Amodio G., Gregori S. (2021). Aging, inflammation and DNA damage in the somatic testicular niche with idiopathic germ cell aplasia. Nat. Commun..

[38] Shi G., Bai Y., Zhang X., Su J., Pang J., He Q., Zeng P., Ding J., Xiong Y., Zhang J. (2022). Bend family proteins mark chromatin boundaries and synergistically promote early germ cell differentiation. Protein Cell.

[39] Artoni F., Kreipke R.E., Palmeira O., Dixon C., Goldberg Z., Ruohola-Baker H. (2017). Loss of *foxo* rescues stem cell aging in *Drosophila* germ line. eLife.

[40] Herati A.S., Zhelyazkova B.H., Butler P.R., Lamb D.J. (2017). Age-related alterations in the genetics and genomics of the male germ line. Fertil. Steril..

[41] Brunet A., Goodell M.A., Rando T.A. (2023). Ageing and rejuvenation of tissue stem cells and their niches. Nat. Rev. Mol. Cell Biol..

[42] Jasper H. (2020). Intestinal Stem Cell Aging: Origins and Interventions. Annu. Rev. Physiol..

[43] Hong X., Campanario S., Ramírez-Pardo I., Grima-Terrén M., Isern J., Muñoz-Cánoves P. (2022). Stem cell aging in the skeletal muscle: The importance of communication. Ageing Res. Rev..

[44] Sênos Demarco R., Uyemura B.S., D’Alterio C., Jones D.L. (2019). Mitochondrial fusion regulates lipid homeostasis and stem cell maintenance in the Drosophila testis. Nat. Cell Biol..

[45] Chandrasekhara C., Ranjan R., Urban J.A., Davis B.E.M., Ku W.L., Snedeker J., Zhao K., Chen X. (2023). A single N-terminal amino acid determines the distinct roles of histones H3 and H3.3 in the Drosophila male germline stem cell lineage. PLoS Biol..

[46] Dolezal D., Liu Z., Zhou Q., Pignoni F. (2015). Fly LMBR1/LIMR-type protein Lilipod promotes germ-line stem cell self-renewal by enhancing BMP signaling. Proc. Natl. Acad. Sci. USA.

[47] Michel M., Raabe I., Kupinski A.P., Pérez-Palencia R., Bökel C. (2011). Local BMP receptor activation at adherens junctions in the Drosophila germline stem cell niche. Nat. Commun..

[48] Rahman M.S., Akhtar N., Jamil H.M., Banik R.S., Asaduzzaman S.M. (2015). TGF-β/BMP signaling and other molecular events: Regulation of osteoblastogenesis and bone formation. Bone Res..

[49] Akiyama T., Gibson M.C. (2015). Decapentaplegic and growth control in the developing Drosophila wing. Nature.

[50] Du G., Xiong L., Li X., Zhuo Z., Zhuang X., Yu Z., Wu L., Xiao D., Liu Z., Jie M. (2020). Peroxisome Elevation Induces Stem Cell Differentiation and Intestinal Epithelial Repair. Dev. Cell.

