# Supplementary material for: Adar Regulates Drosophila melanogaster Spermatogenesis via Modulation of BMP Signaling
Source: Int J Mol Sci. 2024 May 22;25(11):5643. doi: 10.3390/ijms25115643 (PMC11171878; doi:10.3390/ijms25115643)
Supplement: Supplementary file 1 [file ijms-25-05643-s001.zip › Supplementary_Material-revised version.pdf]

Supplementary Figures

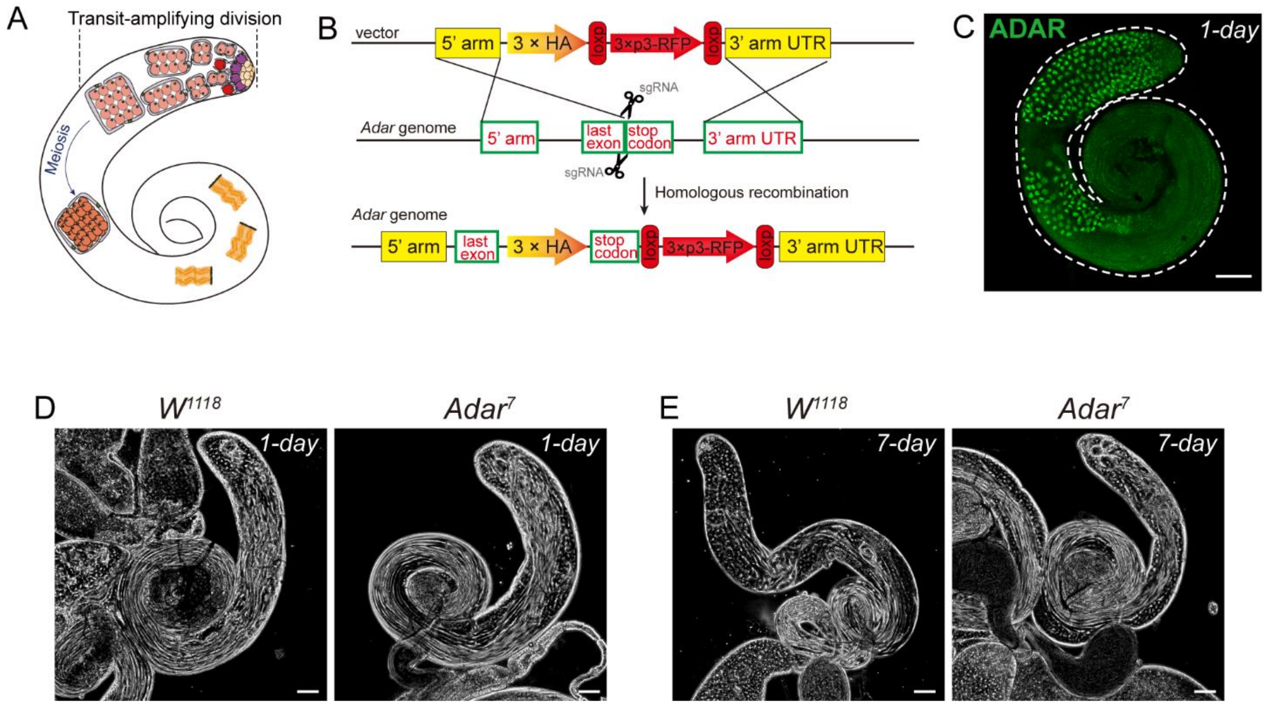

Figure S1. ADAR expression in germline cells and its mutant morphology. (**A**) Schematic diagram of testis tissue in *Drosophila.* (**B**) The approach utilized for generating the endogenous *Drosophila* ADAR-HA knock-in line entailed applying the CRISPR/Cas9 system. For details, please refer to the methods. (**C**) Representative image of ADAR (green) in the testis of 1-day flies. Scale bars represented 100 μm. (**D** and **E**) Testes of wildtype and *Adar* mutants. Scale bars represented 75 μm.

.

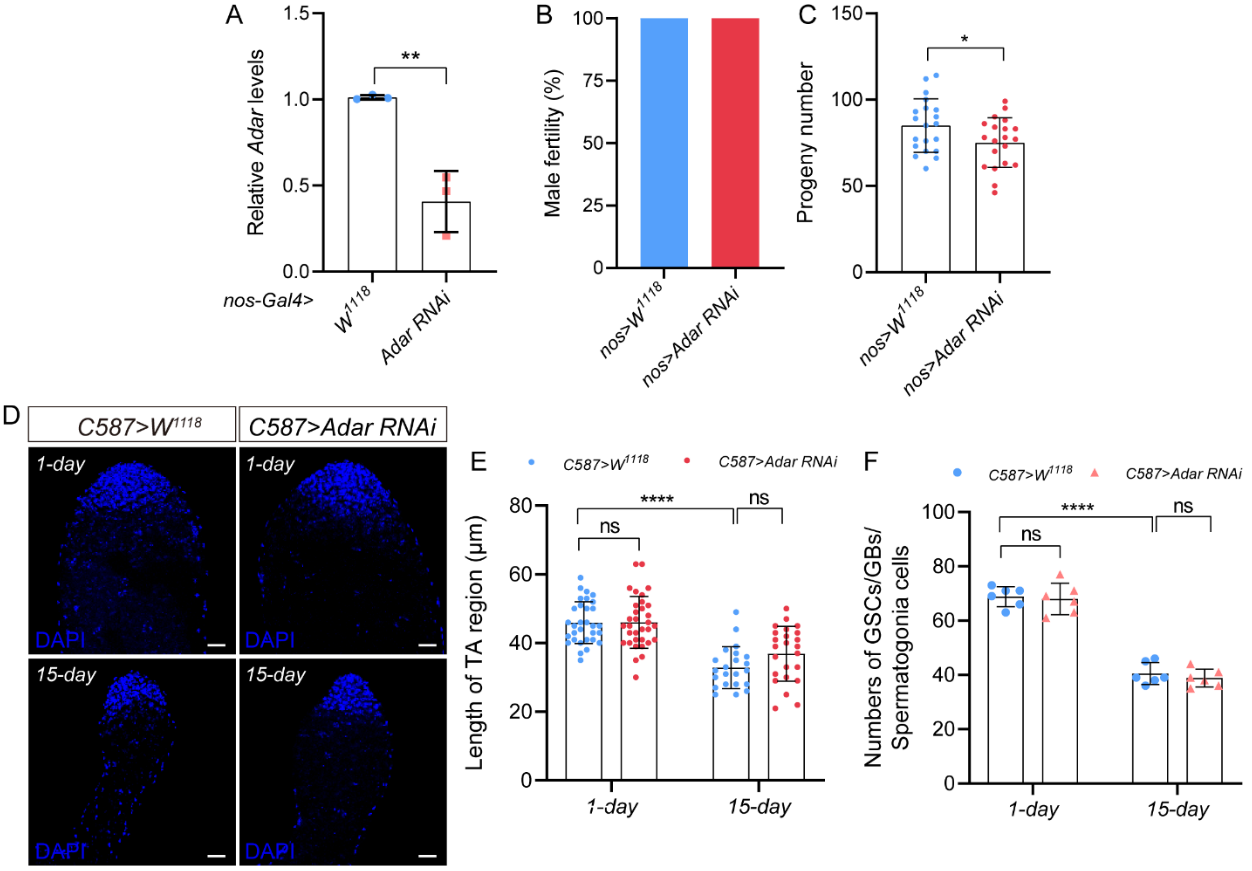

Figure S2. Depletion of ADAR in GSC leads to the accumulation of transit-amplifying germline cells in *Drosophila* testis. (**A**) *Adar* mRNA levels in flies carrying *nos-Gal4*-driven *Adar RNAi.* The expression in the control group was normalized to 1 (n = 3 biologically independent samples). (**B** and **C**) Rate of male fertility and mean number of progeny for flies of different genotypes. (**D**) Immunofluorescence images of testis carrying *C587-Gal4*-driven *W*^{1118} (control) and *Adar RNAi*. DAPI-stained (blue) bright cells were TA regions. Scale bars represented 20 μm. (**E**) Quantification of the length of TA regions in the testis of flies carrying *C587-Gal4*-driven *W*^{1118} (control) and *Adar RNAi*. Each dot represents a testis. (**F**) Quantifying the number of GSCs, GBs, and spermatogonia in different genotypes of *Drosophila*. *, p <0.05; **, p <0.01; ****, p <0.0001, ns, p >0.05.

.

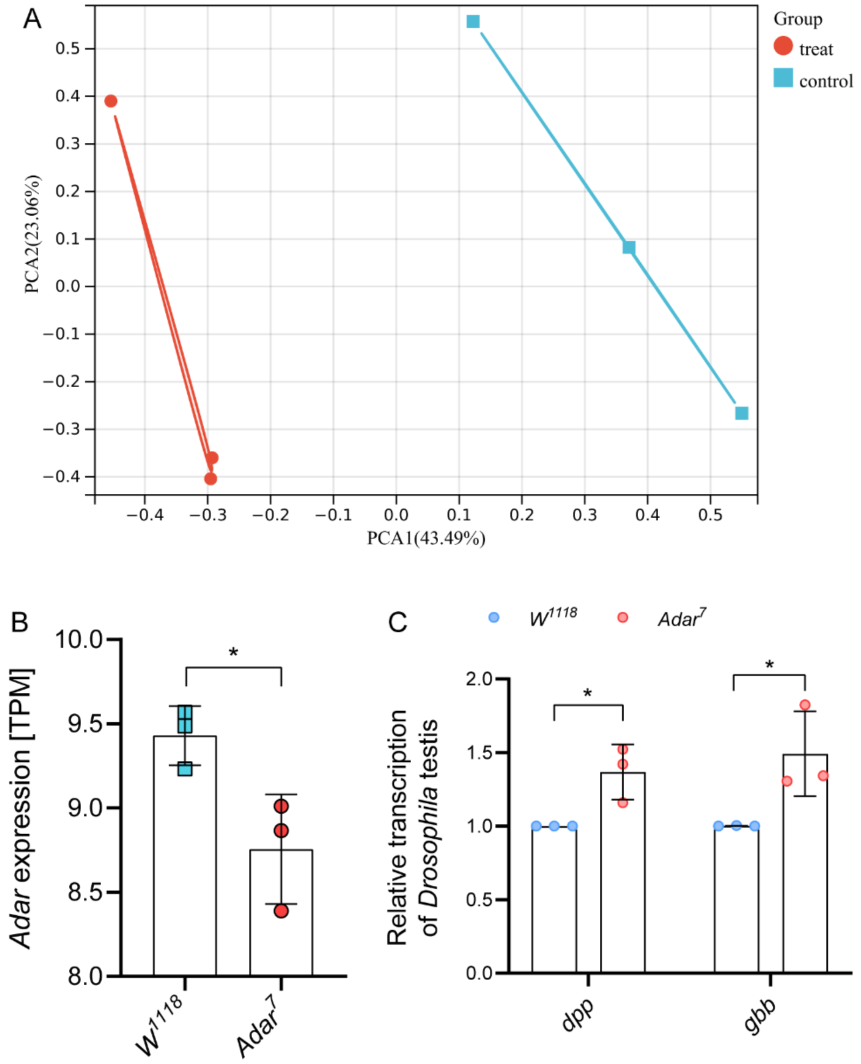

Figure S3. *Adar* regulates male germline stem and progenitor cell differentiation through modulation of BMP signaling. (**A**) Principal component analysis (PCA) of RNA-seq data. The control group indicated W^{1118}, and the treat group indicated $Adar^{7}$. (**B**) TPM (Transcripts Per Kilobase Million) values of *Adar* RNA expression in the RNA-seq data from 15-day W^{1118} (control) and $Adar^{7}$ (n = 3 biologically independent samples). (**C**) Relative mRNA fold changes of *dpp* and *gbb* in 15-day W^{1118} (control) and $Adar^{7}$. The expression in the control group was normalized to 1 (n = 3 biologically independent samples). *, p <0.05.
